# Lariat preparation using a snare catheter for removal of a pancreaticogastric stent in a rendezvous stent exchange

**DOI:** 10.1055/a-2058-8265

**Published:** 2023-04-26

**Authors:** Yoshihide Kanno, Haruka Okano, Fumisato Kozakai, Shinsuke Koshita, Takahisa Ogawa, Toshitaka Sakai, Kei Ito

**Affiliations:** Department of Gastroenterology, Sendai City Medical Center, Sendai, Japan

A 61-year-old man who had previously undergone pancreaticoduodenectomy underwent subsequent endoscopic ultrasound (EUS)-guided stent placement to bridge the jejunum, pancreatic duct, and stomach as treatment for pancreaticojejunal anastomosis stricture ( Fig. 1 ). Stent exchange was attempted 6 months later ( Video 1 ). However, a guidewire could not be advanced along or through the previous stent from the stomach due to an unstable scope position.

**Fig. 1:**
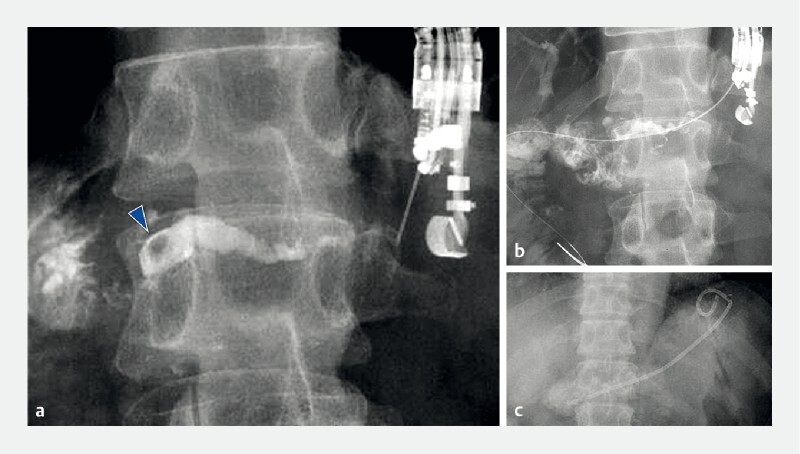
Previous endoscopic ultrasound-guided pancreatic duct drainage. **a** Contrast injection revealed an anastomotic stricture and a stone inside the main pancreatic duct (arrowhead). **b** Guidewire insertion. **c** A 7-Fr single-pigtail stent was deployed bridging the jejunum, pancreatic duct, and stomach.

**Video 1** Previous endoscopic ultrasound-guided pancreatic duct drainage and stent exchange using the lariat method.


A lariat was prepared to facilitate safe removal of the stent during endoscopic intervention from the jejunum where the pancreatic duct had been anastomosed. The wire tip of a snare catheter was grasped using forceps inserted through a single-balloon enteroscope ( Fig. 2 ), and the scope and grasped catheter were then inserted into the stomach. After the catheter was released, the stent was caught and held with the snare like a lariat ( Fig. 3 a, b ).

**Fig. 2:**
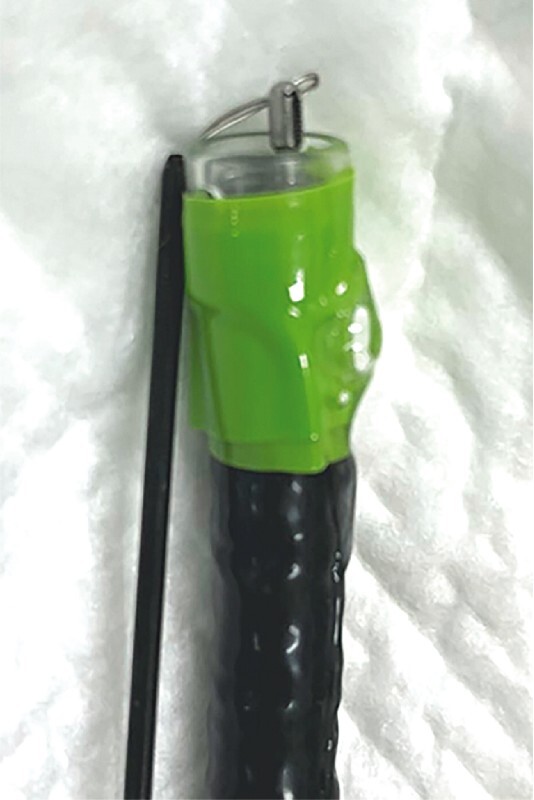
Preparation of the lariat. The wire tip of a snare catheter was grasped using forceps, which was inserted through the working channel of a single-balloon enteroscope with hood attachment.

**Fig. 3:**
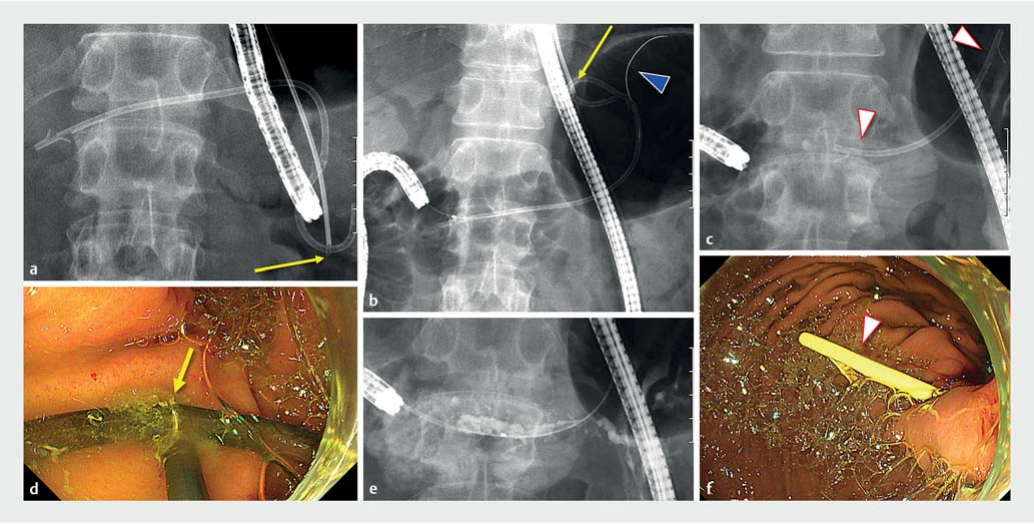
Stent exchange with support of the lariat method. **a, b** The previously deployed stent was caught and held using the lariat snare (yellow arrows) in the stomach. **c, d** The enteroscope alone was advanced into the jejunum and a guidewire (blue arrowhead) was inserted from the jejunum to the stomach through the stent. **e, f** After balloon dilation of the stricture and stone removal, a 7-Fr, 7-cm straight stent (red arrowheads) was deployed bridging the pancreatic duct and stomach.

The scope was subsequently advanced into the jejunum ( Fig. 3 c ). Then, after a guidewire was inserted from the jejunum to the stomach through the stent ( Fig. 3 d ), the stent was removed by withdrawing the snare catheter from the mouth. Following several procedures, a straight stent was deployed between the pancreatic duct and stomach ( Fig. 3 e, f ).

Endoscopic observation during scope withdrawal confirmed that a few centimeters of the stent were located in the stomach.

If the lariat method is not used, there are few strategies by which the previous stent can be safely removed while retaining guidewire routes 1 2 . If two endoscopy systems are available so that double endoscopes can be used, the stent can be removed using forceps via an endoscope at the stomach while a route-keeping guidewire is retained through the other endoscope at the jejunum. However, two endoscopy systems are cumbersome for ordinary endoscopy units to prepare, and insertion of two endoscopes is burdensome. The presented lariat method can be applied with secure maintenance of an access route at ordinary institutions.

Endoscopy_UCTN_Code_TTT_1AS_2AD

## Notes

Endoscopy E-Videos

https://eref.thieme.de/e-videos
*E-Videos* is an open access online section of the journal *Endoscopy* , reporting on interesting cases and new techniques in gastroenterological endoscopy. All papers include a high-quality video and are published with a Creative Commons CC-BY license. Endoscopy E-Videos qualify for HINARI discounts and waivers and eligibility is automatically checked during the submission process. We grant 100% waivers to articles whose corresponding authors are based in Group A countries and 50% waivers to those who are based in Group B countries as classified by Research4Life (see: https://www.research4life.org/access/eligibility/ ). This section has its own submission website at https://mc.manuscriptcentral.com/e-videos
